# Clinicopathological Factors Influencing Lymph Node Yield in Colorectal Cancer: A Retrospective Study

**DOI:** 10.1155/2019/5197914

**Published:** 2019-01-22

**Authors:** Elena Orsenigo, Giulia Gasparini, Michele Carlucci

**Affiliations:** ^1^Department of General and Emergency Surgery, San Raffaele Scientific Institute, 20132 Milan, Italy; ^2^Vita-Salute San Raffaele University, 20132 Milan, Italy

## Abstract

Many colorectal resections do not meet the minimum of 12 lymph nodes (LNs) recommended by the American Joint Committee on Cancer for accurate staging of colorectal cancer. The aim of this study was to investigate factors affecting the number of the adequate nodal yield in colorectal specimens subject to routine pathological assessment. We have retrospectively analysed the data of 2319 curatively resected colorectal cancer patients in San Raffaele Scientific Institute, Milan, between 1993 and 2017 (1259 colon cancer patients and 675 rectal cancer patients plus 385 rectal cancer patients who underwent neoadjuvant therapy). The factors influencing lymph node retrieval were subjected to uni- and multivariate analyses. Moreover, a survival analysis was carried out to verify the prognostic implications of nodal counts. The mean number of evaluated nodes was 24.08 ± 11.4, 20.34 ± 11.8, and 15.33 ± 9.64 in surgically treated right-sided colon cancer, left-sided colon cancer, and rectal tumors, respectively. More than 12 lymph nodes were reported in surgical specimens in 1094 (86.9%) cases in the colon cohort and in 425 (63%) cases in the rectal cohort, and patients who underwent neoadjuvant chemoradiation were analysed separately. On univariate analysis of the colon cancer group, higher LNs counts were associated with female sex, right colon cancer, emergency surgery, pT3-T4 diseases, higher tumor size, and resected specimen length. On multivariate analysis right colon tumors, larger mean size of tumor, length of specimen, pT3-T4 disease, and female sex were found to significantly affect lymph node retrieval. Colon cancer patients with 12 or more lymph nodes removed had a significantly better long-term survival than those with 11 or fewer nodes (*P* = 0.002, log-rank test). Rectal cancer patients with 12 or more lymph nodes removed approached but did not reach a statistically different survival (*P* = 0.055, log-rank test). Multiple tumor and patients' factors are associated with lymph node yield, but only the removal of at least 12 lymph nodes will reliably determine lymph node status.

## 1. Introduction

Examination of an adequate number of lymph nodes (LNs) is a decisive factor for the correct staging and subsequent therapy for colorectal cancer (CRC) patients. The presence of metastatic lymph nodes represents a step toward systemic tumor spread and it is therefore a strong indicator of adverse prognosis [1], and node metastasis is the major determinant of adjuvant therapy for patients with CRC. In 2007, the American Joint Commission on Cancer and the National Quality Forum endorsed the harvest of 12 lymph nodes as a standard quality indicator for CRC resection specimens [2, 3]; moreover, a higher number of sampled lymph nodes has emerged as an independent prognostic factor for improved survival in several previous studies [4–7]—but data are still conflicting [8]. There are different factors that can affect node retrieval and can be classified as surgeon-, pathologist-, disease-, and patient-related. Both patient- and disease-related variables are nonmodifiable and pose the question of whether the minimum number of examined LNs must be individually assigned. End points of the study were to explore the link between compliance with the ≥12-node cut-off and different variables, such as age, sex, BMI, tumor characteristics, and type of surgery.

## 2. Materials and Methods

All patients undergoing colorectal resections between 1993 and 2016 at our surgical center were reviewed to identify colorectal resections carried out for colorectal cancer. Patients operated for nonmalignant conditions were excluded. All data were prospectively collected and recorded in a database. The factors potentially affecting the number of lymph nodes identified in surgical specimens were analysed retrospectively, including demographic data, pathologic features of the tumor, and patient survival. The study was approved by the local Bioethics Committee. The determination of the final number of lymph nodes examined was based exclusively on the final pathologic report. Specimens were examined by the Pathology Department according to the 7th edition of the AJCC/UICC TNM classification [9]. Lymph nodes were identified by haematoxylin and eosin staining. Patient data were analysed separately for colon (1259 patients) and rectal cancer (675 patients) patients. Tumors located in the rectum or at the rectosigmoid junction were summarized as rectal cancers. Tumors originating from the sigmoid colon to the left colonic flexure were defined as left-sided cancers, while tumors located from the transverse colon to the caecum were defined as right-sided cancers. We excluded patients with T1 cancer treated by endoscopic polypectomy. We also excluded 385 patients who received neoadjuvant therapy, since the number of harvested lymph nodes might be influenced by the neoadjuvant therapy [6], and we analysed them as a separate cohort. Study variables included age, gender, BMI, TNM stage, resected specimen length, cancer site, and type of surgery (laparoscopic vs. open surgery, emergency vs. elective surgery). Preliminary analyses used descriptive statistics to summarize the demographic characteristics of patients. Univariate analyses using the *χ*^2^ test, Spearman's rho, and an independent *t* test were undertaken to explore the relationship between selected factors and the reported presence of at least 12 lymph nodes. ROC curve and Youden index were used to determine optimal cut-offs. Binary logistic regression model was applied to assess the influence of primary tumor characteristics on the number of retrieved lymph nodes. Statistical analysis was performed using SPSS, version 20.0, software package (SPSS Inc., Chicago, IL).

## 3. Results and Discussion

The patients' characteristics are shown in Tables 1, 2, and 3. The mean number of evaluated nodes was 24.08 ± 11.4, 20.34 ± 11.8, and 15.33 ± 9.64 in surgically treated right-sided colon cancer, left-sided colon cancer, and rectal tumors, respectively. Mean surgical specimen length was 26.3 cm in the colon cohort and 21 cm in the rectal cohort. A cut-off of 3.5 cm for tumor dimension and of 20 cm for specimen length was set in the colon cohort. A cut-off of 3.7 cm for tumor size and of 15 cm for specimen length was set in the rectal cohort.

### 3.1. Colon Cancer Patients

**Figure 1:**
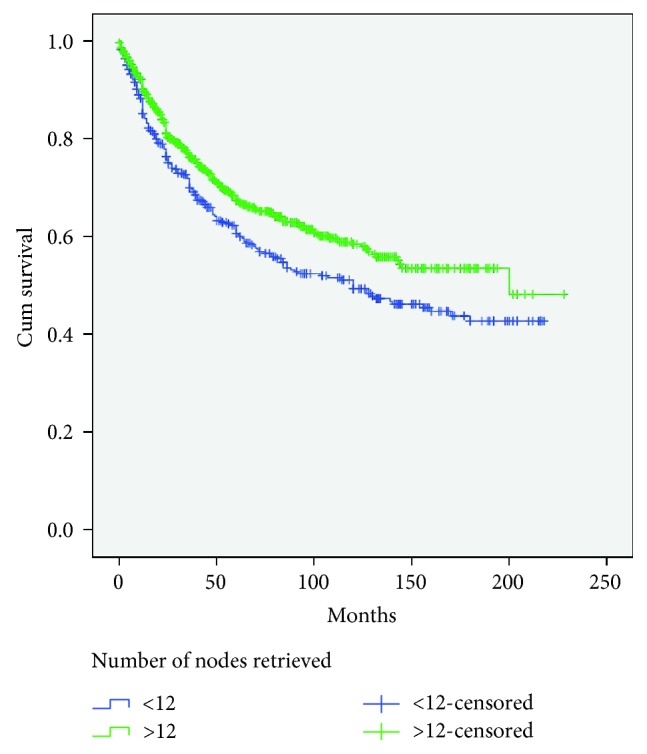
Kaplan-Meier survival curves for colon cancer according to the number of evaluated lymph nodes. Patients with 12 or more lymph nodes removed had a significantly better long-term survival than those with 11 or fewer nodes (*P* = 0.002, log-rank test).

Based on univariate analysis, a higher LN count had a relationship with female sex (*P* = 0.02), right colon cancer (*P* < 0.001), emergency surgery (*P* = 0.007), pT3-T4 diseases (*P* < 0.001), higher tumor dimension (*P* < 0.001), and resected specimen length (*P* < 0.001). No significant difference was seen in terms of age, node positivity, laparoscopic or open surgery, and BMI. Using a multivariate logistic regression analysis, the high-harvest group was significantly associated with right colon tumors (*P* ≤ 0.001; odds ratio (OR), 0.580; 95% confidence interval (CI), 0.409-0.823), larger tumor mean size (*P* < 0.001; OR, 3.371; 95% CI, 2.238-5.077), longer resected specimen (*P* < 0.001; OR, 2.192; 95% CI, 1.502-3.199), pT3-T4 disease (*P* < 0.001; OR, 1.495; 95% CI, 1.192-1.876), and female sex (*P* < 0.001; OR, 1.898; 95% CI, 1.285-2.804) (Table 4). In colon cancer patients, retrieval of less than 12 lymph nodes had a negative effect on patients' survival (log rank: *P* = 0.002) (Figure 1).

### 3.2. Rectal Cancer Patients

**Figure 2:**
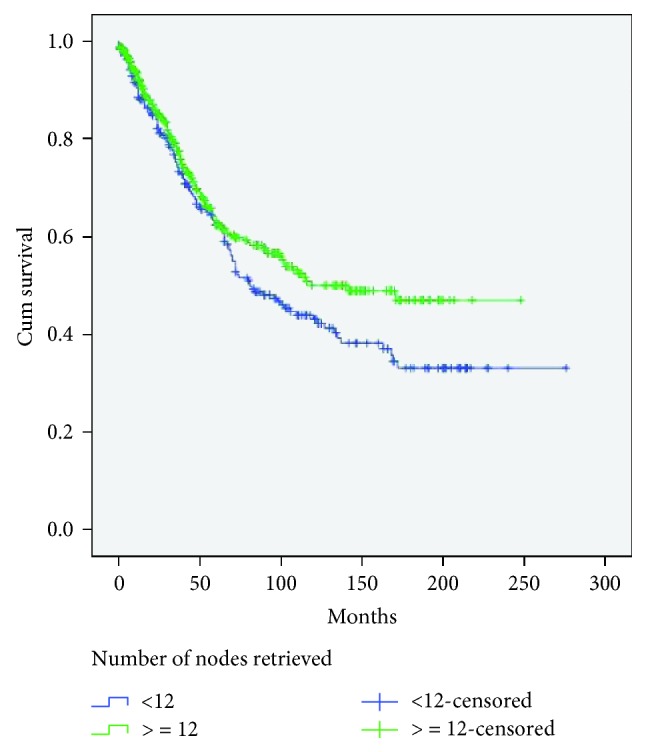
Kaplan-Meier survival curves for rectal cancer without neoadjuvant treatment according to the number of evaluated lymph nodes. Patients with 12 or more lymph nodes removed had reduced long-term survival than those with 11 or fewer nodes, approaching but not reaching statistical significance (*P* = 0.055, log-rank test).

Table 5 shows the correlations between clinicopathological features and harvested lymph nodes for rectal cancer patients. Using univariate analysis, it was demonstrated that there were statistically significant differences between the two groups in terms of tumor size (*P* < 0.001), resected specimen length (*P* < 0.001), T stage (*P* < 0.001), emergency surgery (*P* < 0.023), and node positivity (*P* < 0.001). There were no significant differences with age, sex, and BMI. On multivariate logistic regression analysis, mean tumor size was significantly larger for the high-harvest group than for the low-harvest group (*P* < 0.001; OR, 2.061; 95% CI, 1.456-2.919), and specimen length (*P* < 0.001; OR 2.210; 95% CI, 1.501-3.255) and T stage (*P* < 0.005; OR 1.456; 95% CI, 1.148-1.847) were statistically significant. In the rectal cancer cohort, patients who had less than 12 lymph nodes retrieved had a reduced survival, although it did not reach statistical significance (log rank: *P* = 0.055) (Figure 2).

**Figure 3:**
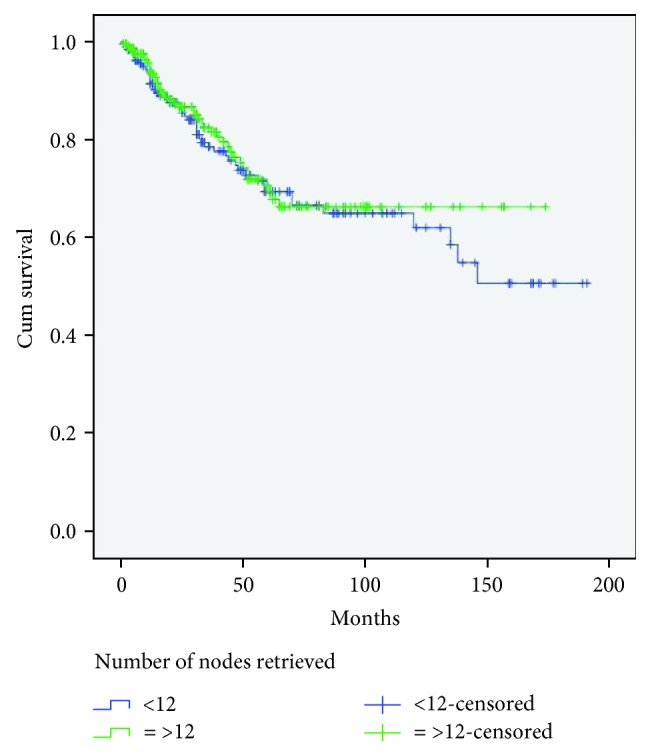
Kaplan-Meier survival curves for rectal cancer with neoadjuvant treatment according to the number of evaluated lymph nodes. Patients with 12 or more lymph nodes removed had similar long-term survival than those with 11 or fewer nodes (*P* = 0.575, log-rank test).

In Table 6, we show the correlations between clinicopathological features and harvested lymph nodes for rectal cancer patients who underwent neoadjuvant therapy. The only statistically significant correlation is with specimen length (*P* = 0.017; OR, 2.210; 95% CI, 1.501-3.255). Moreover, in the rectal cohort who underwent neoadjuvant treatment, the number of lymph nodes yielded was statistically significantly lower than in the non-neoadjuvated cohort (*P* < 0.003; OR, 0.676; 95% CI, 0.524-0.872). In rectal cancer patients who underwent neoadjuvant treatment, removal of less than 12 lymph nodes retrieved had no effect on patients' survival (log rank: *P* = 0.575) (Figure 3).

## 4. Conclusions

The prognosis of patients with CRC after tumor resection is mainly defined by the presence of neoplastic cells in lymph nodes. The number of sampled and histologically analysed LNs has therefore a fundamental role, not only as an independent prognostic marker for therapeutic decisions but also as a marker for adequate staging, quality of surgery, and pathologic analysis [10, 11]. How to obtain adequate lymph nodes remains an important issue in colorectal cancer. According to our data, retrieval of more than 12 LNs was associated with tumor size and specimen length in both univariate and multivariate analyses. Tumor size is an established predictor of LNs yield [8, 12–16] that was confirmed by our study. Larger tumors may be more visible on pathologic examination due to increased cancer antigen and inflammation response. It has been proposed that larger tumors elicit an intense antigenic immune response within the regional LNs basin, making them more visible to pathologic examination and possibly leading to increasing LNs yields [13, 15]. This study confirmed surgical length to be an independent predictor of LNs number; in all tumor localizations, longer specimens have significantly more LNs [8, 13, 17–19]. Tumor location by anatomic site also influenced LNs harvest; higher LNs numbers were observed in right colon cancer, even after adjustment for specimen length. This may be explained by variant lymphatic anatomy (i.e., a disproportionate number of LNs exist along the ileocolic artery, and there is a natural decline in LN numbers with more distal progression within the colonic mesentery) [15] and other variations in tumor biology, such as microsatellite instability. Differences in embryonic development or a greater length of the mesenteric root have been discussed as a possible causes [16]. However, also a higher inflammatory response to right-sided tumors, which are often microsatellite instable, has been proposed and found in previous analyses [20–22]. Age impacted significantly on LNs yield in the rectal cohort, as several other studies have demonstrated. This phenomenon may result from a complex interplay of patient and surgeon factors, such as older patients are less likely to undergo extensive surgery and their immunological response to cancer is less intense so that LNs might not be visible to the surgeon (and the pathologist) [13, 14, 23]. In the colon cohort, male sex was associated with reduced LNs yield as previously described [24, 25]; moreover, patients undergoing emergency surgery had higher LNs count [26, 27]. In the rectal cohort, laparoscopic surgery was associated with higher lymph node harvest even if it did not reach statistical significance [28–30]. Moreover, neoadjuvant treatment had a significant effect on the number of lymph nodes harvested [31].

The limitations of this study were as follows: it consisted of a retrospective cohort study involving only a single institute and it involved many surgeons. In conclusion, this study demonstrated that only few independent factors were associated with the likelihood of removing at least 12 lymph nodes in surgical specimens of patients undergoing colorectal resection for cancer, i.e., tumor size, T stage, specimen length, right-sided location, and female (colon cancer) were independent factors associated with the number of lymph node retrieval. Patients with 12 or more lymph nodes removed had a significantly better long-term survival than those with 11 or fewer nodes (*P* = 0.002, log-rank test) only in the colon cancer group. In rectal cancer, this factor had no effect on survival, particularly in the neoadjuvated cohort.

## Figures and Tables

**Table 1:** Clinicopathological data of 1259 surgically treated colon cancer patients.

	*n*	%
Gender		
Males	705	56
Females	554	44
Age		
<65	450	35.7
≥65	809	64.3
pT		
T1	153	12.1
T2	154	12.2
T3	741	58.9
T4	211	16.8
Laterality		
Right colon	610	48.5
Left colon	622	49.4
Total colectomy	27	2.1
Harvested LN		
<12	1096	87.1
≥12	163	12.9
Mean LN (SD)		
22.53 (13.13)		

LNs: lymph nodes; pT: pathological tumor stage.

**Table 2:** Clinicopathological data of 675 surgically treated rectal cancer patients without neoadjuvant therapy.

	*n*	%
Gender		
Males	426	63.1
Females	249	36.9
Age		
<65	340	50.4
≥65	335	49.6
pT		
T1	94	13.9
T2	150	22.2
T3	379	56.1
T4	52	7.7
Harvested LN		
<12	250	37
≥12	425	63
Mean LN (SD)		
15.33 (9.64)	—	—

LNs: lymph nodes; pT: pathological tumor stage.

**Table 3:** Clinicopathological data of 385 surgically treated rectal cancer patients who underwent neoadjuvant therapy.

	*n*	%
Gender		
Males	244	63.4
Females	141	36.6
Age		
<65	237	63.4
≥65	148	36.6
pT		
Complete remission	68	17.5
T1	25	6.5
T2	88	22.7
T3	179	46.7
T4	25	7.7
Harvested LN		
<12	182	47.3
≥12	203	52.7
Mean LN (SD)		
12.87 (8.07)	—	—

**Table 4:** Univariate and multivariate logistic regression analyses of clinicopathological factors influencing lymph node retrieval in 1259 colon cancer specimens.

Factor	Univariate			Multivariate		
	*P* value	95% CI	OR	*P* value	95% CI	OR
Gender	0.021	1.063-2.107	1.497	≤0.001	1.285-2.804	1.898
Specimen length	≤0.001	1.014-1.051	1.032	≤0.001	1.502-3.199	2.192
Emergency surgery	≤0.001	1.256-3.681	2.150	0.269	0.760–2.674	1.426
Tumor location	≤0.001	0.414-0.761	0.561	0.002	0.409-0.823	0.580
T status	≤0.001	1.682-2.404	2.011	≤0.001	1.192-1.876	1.495
N status	0.023	1.040-1.671	1.318	0.781	0.779-1.395	1.042
Tumor dimension	≤0.001	3.246-6.778	4.691	≤0.001	2.238-5.077	3.371

OR: odds ratio; CI: confidence interval.

**Table 5:** Univariate and multivariate logistic regression analyses of clinicopathological factors influencing lymph node retrieval in 675 rectal cancer specimens.

Factor	Univariate			Multivariate		
	*P* value	95% CI	OR	*P* value	95% CI	OR
Specimen length	≤0.001	2.025-4.165	2.904	≤0.001	1.501-3.255	2.210
Emergency surgery	0.023	1.092-3.247	1.883	0.133	0.874-2.777	1.558
N positivity	≤0.001	1.471-2.824	2.038	0.287	0.793-2.184	1.317
T status	≤0.001	1.636-2.443	1.999	0.002	1.148-1.847	1.456
N status	≤0.001	1.089-1.573	1.308	0.902	0.784-1.318	1.016
Tumor dimension	≤0.001	2.031-3.873	2.804	≤0.001	1.456-2.919	2.061

OR: odds ratio; CI: confidence interval.

**Table 6:** Univariate and multivariate logistic regression analyses of clinicopathological factors influencing lymph node retrieval in 385 rectal cancer specimens of patients who underwent neoadjuvant treatment.

Factor	Univariate			Multivariate		
	*P* value	95% CI	OR	*P* value	95% CI	OR
Specimen length	≤0.001	1.027-1.092	1.059	0.017	1.119-3.158	2.321
Emergency surgery	0.391	1.025-2.254	0.531	0.093	0.054-1.249	0.261
N positivity	0.002	1.277-2.984	1.952	0.245	0.748-3.122	1.528
T status	0.003	1.089-1.529	1.290	0.319	0.905-1.357	1.108
N status	0.011	1.086-1.865	1.423	0.777	0.677-1.686	1.068
Tumor dimension	≤0.002	1.367-4.186	2.392	0.009	1.232-3.122	2.321

OR: odds ratio; CI: confidence interval.

## Data Availability

The clinical data used to support the findings of this study are restricted by the San Raffaele IRCCS Ethic Committee in order to protect patients' privacy. Data are available from the corresponding author Dr. Elena Orsenigo (orsenigo.elena@hsr.it) for researchers who meet the criteria for access to confidential data.
